# Plate Food Waste in Early Childhood Education: Contextual and Nutritional Drivers with Implications for Sustainable Food Systems

**DOI:** 10.3390/foods14203545

**Published:** 2025-10-17

**Authors:** Dimitrie Stoica, Monica Laura Zlati, Raisa Bălan (Stanciu), Carmelia Mariana Bălănică Dragomir, Cezar Ionuț Bichescu, Florentina-Loredana Dragomir-Constantin, Maricica Stoica

**Affiliations:** 1 Department of Applied Sciences, Cross-Border Faculty, “Dunarea de Jos” University of Galati, 111 Domneasca Street, 800201 Galati, Romania; dimitrie.stoica@ugal.ro (D.S.); carmelia.dragomir@ugal.ro (C.M.B.D.); 2 Department of Business Administration, Faculty of Economics and Business Administration, “Dunarea de Jos” University of Galati, 59-61 Balcescu Street, 800001 Galati, Romania; 3 Department of Life Sciences, Cross-Border Faculty, “Dunarea de Jos” University of Galati, 111 Domneasca Street, 800201 Galati, Romaniacezar.bichescu@ugal.ro (C.I.B.); 4 Teaching Department, “Andrieș” Kindergarten, Vadul lui Isac Village, MD-5321 Cahul, Moldova; 5 Department of Information Systems and Cyber Operations, National Defense University Carol I, 050662 Bucharest, Romania; dragomir.loredana@unap.ro

**Keywords:** plate food waste, early childhood education, kindergarten meals, portion control, menu design, serving strategy, nutrition policy, sustainability

## Abstract

Plate food waste (PFW) in early childhood education is a critical yet understudied issue in Eastern Europe, with implications for nutrition, sustainability, and food security. This study examined PFW in a kindergarten in the Republic of Moldova, encompassing all 58 enrolled children and generating 14,292 meal-level observations through direct weighing of served meals and leftovers. Variance analysis (ANOVA) was used to test the influence of weekday, meal type, age, and gender, while Principal Component Analysis (PCA) explored latent structures of waste determinants. Results showed significant effects of weekday and meal type on PFW, with lunch consistently generating the highest waste levels and snacks the lowest. Gender differences were modest, while the interaction between age and gender indicated heterogeneous developmental patterns in waste behavior. PCA reduced the dataset to three main components: Portion Control, Menu Design, and Serving Strategy, explaining 84.7% of the total variance. These findings provide novel evidence for understanding how contextual and nutritional variables shape children’s PFW in early education and offer a replicable framework for reducing PFW and improving dietary adequacy in kindergartens. The study’s implications extend to sustainable nutrition planning and early behavioral interventions in preschool settings.

## 1. Introduction

Food waste (FW) has become one of the defining sustainability challenges of the 21st century, exerting significant environmental, social, and economic impacts worldwide. Estimates indicate that nearly one-third of food produced for human consumption is lost or wasted annually, contributing 8–10% of global greenhouse gas emissions and placing severe pressure on freshwater, land, and biodiversity through excessive use of agricultural inputs and energy resources [1,2,3,4,5,6]. Beyond its environmental footprint, food waste (food suitable for human consumption, but is not eaten) undermines food security, intensifies economic inequality, and complicates the achievement of the Sustainable Development Goals [7,8]. The urgency of addressing this issue has led international organizations and national governments to call for systematic interventions along the entire supply chain, with a particular emphasis on the consumption stage where FW are most prevalent [9]. In high- and middle-income economies, the most FW occurs during the consumption phase, such as in households, private food services (HoReCa—Hotel Restaurant Catering), and public catering institutions (schools, canteens, hospitals), whereas in low-income regions, food losses are predominantly concentrated during production and distribution due to infrastructural and logistical constraints [10,11,12,13,14,15].

In food service settings, FW can occur at multiple points along the service chain: during food preparation in the kitchen, through handling by staff or educators, and ultimately as plate waste (PFW) left uneaten by consumers.

Research has highlighted the role of consumer behaviors, menu design, and institutional practices in shaping waste patterns, with growing evidence from the HoReCa sector and higher education contexts [12,16,17]. However, early childhood education (ECE) environments, including kindergartens, remain understudied despite their dual relevance: as sites of significant food provision and as formative settings where children’s lifelong food preferences and behaviors begin to take shape [18].

The Republic of Moldova provides a particularly relevant case study. Despite strict national catering standards for preschool institutions, recent assessments estimate that nearly 180,000 tonnes of food are wasted annually, corresponding to a FW index of 93.4/100 [19]. This article focuses specifically on PFW (the portion of food served in kindergartens that remains uneaten by children), an area for which empirical evidence in the Republic of Moldova is virtually absent. Recent studies have explored FW generation in educational settings, showing that a substantial proportion of waste occurs directly on children’s plates. For instance, Kasavan et al. [20] found that approximately 17% of total food prepared in Malaysian school canteens became waste, while Filimonau et al. [21] reported that public kindergartens in Russia generated more waste than private ones due to limited flexibility and parental engagement. Similarly, Nguyen et al. [22] observed that primary school students in Vietnam discarded about 23% of food served during lunch, with vegetables being the most wasted items. Although global awareness of FW has expanded rapidly, research evidence from Eastern Europe and post-transition economies remains scarce, especially in preschool and early education institutions. By providing primary, systematically collected data from a public kindergarten in the Republic of Moldova, this study contributes new empirical insights into an underexplored geographical and institutional context.

Understanding how contextual (e.g., day of the week, type of meal) and nutritional (e.g., portion size, macronutrient balance) factors interact with demographic characteristics (age, gender) is essential for designing effective PFW reduction strategies in preschool settings. Reducing PFW in ECE has not only nutritional and educational implications but also a strategic importance. According to FAO [2], minimizing food loss and waste contributes directly to food system resilience and resource efficiency. UNESCO [23] and UNICEF [24] further highlight that early education institutions play a key role in promoting sustainable consumption habits from childhood, thereby supporting food security, social stability, and long-term environmental goals.

Therefore, analyzing the contextual and nutritional factors of PFW in children is not merely a local concern but part of a broader framework of international resilience and security.

Against this backdrop, the present study formulates and empirically tests two hypotheses. First (H1), that PFW in preschool settings is significantly influenced by contextual and nutritional factors such as meal type and day of the week. Second (H2), that demographic characteristics including age and gender exert measurable effects on the amount of PFW. To complement these hypothesis-driven analyses, a principal component analysis (PCA) was conducted in order to identify underlying dimensions that structure waste patterns across the sample. This dual approach not only strengthens the robustness of the findings but also ensures that both surface-level variations and latent determinants are captured.

This study provides an empirical assessment of PFW in an ECE setting in the Republic of Moldova, integrating direct weighing, nutritional composition data, and multivariate analysis. Beyond filling a regional research gap, it contributes methodologically by identifying key latent dimensions of waste generation: portion control, menu design, and serving strategy, relevant for evidence-based policy design in preschool institutions. The present study adopts an exploratory approach aimed at identifying preliminary patterns and contextual determinants of PFW rather than establishing causal relationships. The results are therefore interpreted as indicative trends requiring further validation across multiple sites.

## 2. Literature Review

Food waste (FW) in educational settings has increasingly attracted scientific and policy attention, as schools and kindergartens represent critical environments for promoting sustainable consumption and nutrition literacy among children [23,24]. Beyond its economic and environmental implications, FW in early childhood education (ECE) also reflects behavioral and institutional factors related to portioning, menu design, and eating habits. As early-life food experiences shape long-term consumption behaviors, understanding plate food waste (PFW) in these contexts is essential for designing effective interventions. Empirical studies across Asia and Europe have revealed consistent patterns of FW generation in school environments. In Malaysia, Kasavan et al. [20] quantified PFW in ten school canteens and found that 17.4% of prepared food was discarded, primarily due to oversized portions and limited menu diversity. A follow-up investigation emphasized that behavioral engagement of teachers, students, and food handlers is crucial for preventing waste at the institutional level [25]. In the Russian context, Filimonau et al. [21] reported that public kindergartens produced more FW than private ones, attributing this to weaker parental involvement and reduced operational flexibility. Similarly, in Vietnam, Nguyen et al. [22] observed that approximately 23% of food served in primary schools was wasted, with vegetables contributing most to overall waste levels. Complementary findings from Thailand confirmed that imbalanced meal composition and portion oversizing were primary determinants of waste in school lunch programs [26]. European research has also contributed substantial insights into the behavioral mechanisms of PFW. In Portugal, Martins et al. [27] experimentally demonstrated that portion control and improved serving practices reduced waste without diminishing student satisfaction. Croatian data by Ilić et al. [28] confirmed that children’s food preferences, portion size, and taste aversion were the main predictors of PFW, especially for vegetables. Similar developmental differences were reported in the United States, where Niaki et al. [29] found that younger students discarded significantly more food than older ones. These results collectively indicate that children’s sensory acceptance and developmental stage strongly influence food consumption behavior in institutional contexts. More recent studies have explored the intersection between nutritional quality, environmental impact, and institutional management. In Sweden, Sundin et al. [30] linked PFW composition to carbon footprint and nutrient loss, demonstrating that although staple foods accounted for the largest volume of waste. The study emphasized that FW in schools represents both an ecological and nutritional challenge. Similarly, Guimarães et al. [31] conducted a systematic review and meta-analysis showing that the type of food service and distribution system significantly affect the magnitude of PFW, highlighting the need for tailored, context-specific prevention strategies. Intervention-based research has further confirmed the potential of integrated approaches. In Latvia, Lonska et al. [32] compared three strategies: digital tracking, educational campaigns, and organizational adjustments, and found that sustained reductions in waste required continuous monitoring and feedback. Karpouzis et al. [33], in a systematic review of 18 school-based interventions, demonstrated that nutrition education programs incorporating sustainability components such as gardening, tasting, and cooking activities achieved significant improvements in children’s dietary behavior. Together, these studies suggest that reducing PFW requires both operational adjustments and educational reinforcement within a sustainability-oriented framework. Despite these advances, empirical data from Eastern European and post-transition economies remain limited, particularly within ECE institutions. The present study contributes to filling this gap by providing one of the first systematic assessments of PFW in a public kindergarten in the Republic of Moldova, linking nutritional, contextual, and demographic determinants in a region where such data are largely unavailable.

**H1.**  *Contextual factors*

Previous studies have shown that contextual variables, such as the day of the week or meal type, may influence children’s food intake and PFW. Accordingly, we hypothesize that contextual conditions play a significant role in shaping the quantity and variability of food waste in preschool environments.

**Hypothesis** **H1.** *Contextual factors significantly influence PFW levels in kindergarten settings*.

**H1a.**  *Weekday effects*

Although little evidence is available on weekday differences in kindergarten PFW, this study explores whether the proportion of PFW varies according to weekday. Institutional routines, attendance patterns, and menu rotation often differ across the week, potentially shaping both preparation volumes and children’s appetite or acceptance.

**Hypothesis** **H1a.** *In kindergarten, the proportion of PFW differs significantly across days of the week*.

**H1b.**  *Meal-type differences*

Research consistently demonstrates that PFW is not evenly distributed across meals. Evidence from various institutional food services shows that main meals generally generate the largest share of PFW. For instance, in healthcare settings, lunch accounted for 43–61% of total PFW, followed by dinner (22–35%) and breakfast (15–25%) [34]. Similar trends have been documented in educational contexts: a study on senior high school students in China reported 55.28 g of PFW at lunch compared with 54.24 g at dinner and only 22.39 g at breakfast [17], while research among Colombian university students found that lunch accounted for 49% of PFW, compared with 33% at breakfast [35]. Despite the expanding body of research on PFW in schools, hospitals, and hospitality settings, studies focusing on preschool meal services remain scarce, particularly in Eastern Europe, including the Republic of Moldova, where empirical evidence on PFW among young children is almost absent. Early childhood education settings present unique challenges, as young children’s sensory preferences, motor skills, and nutritional needs differ significantly from those of older students or adult consumers. Building on insights from these related sectors, this study examines whether PFW in kindergarten follows a similar distribution across meals, with lunch representing the highest-risk period for PFW.

**Hypothesis** **H1b.** *In kindergarten, PFW varies according to the type of meal (breakfast, snack, lunch, dinner)*.

**H1c.**  *Interaction between weekday and meal type*

To our knowledge, no previous study has examined how weekday and meal type jointly influence PFW in preschool settings. This exploratory hypothesis addresses this gap, proposing that the impact of weekday on PFW may differ across meals due to variations in menu composition, attendance, and operational routines over the week.

**Hypothesis** **H1c.** *In kindergarten, the interaction between weekday and meal type has a significant effect on the amount of PFW*.

**H2.**  *Child characteristics*

Research in food service and hospitality contexts has shown that socio-demographic variables may shape PFW behavior. Age and gender have been identified as potential determinants of PFW or as factors associated with its underlying causes [12,16,36]. Although most of these findings originate from studies in restaurants and other HoReCa environments, they suggest that demographic features may also be relevant in institutional meal services for young children.

**Hypothesis** **H2.** *Children’s socio-demographic characteristics significantly influence PFW levels in kindergarten settings*.

**H2a.**  *Age differences*

Children’s demographic characteristics have been consistently linked to eating behaviors and PFW. Age is often regarded as a key predictor. Younger children typically display higher levels of food neophobia and limited willingness to try unfamiliar foods, which can lead to greater PFW [37]. With age, repeated exposure to a variety of flavors, enhanced fine motor skills, and greater familiarity with structured mealtimes tend to promote acceptance and self-regulation, potentially lowering PFW [38,39]. Nevertheless, several studies conducted in school and kindergarten settings have reported the opposite tendency, indicating that PFW can increase with children’s age, due to greater autonomy in food choices or selective rejection of specific items [29,40,41]. A recent large-scale study in Italian primary school cafeterias also indicates that children’s emotions and perceptions of meal quality are related to both intake and PFW, highlighting how socio-demographic variables influence the way meals are experienced and consumed [42]. These findings underline the relevance of age as an explanatory variable when investigating the drivers of PFW in early childhood settings.

**Hypothesis** **H2a.** *PFW in kindergarten increases with children’s age*.

**H2b.**  *Gender differences*

Gender has also been discussed as a determinant of PFW. Some research suggests that boys may consume larger portions yet leave more leftovers, particularly for vegetables or foods with complex textures, while girls tend to be more selective in their choices but finish a greater proportion of what they select [43,44,45]. These findings emphasize the relevance of gender as an explanatory factor in studies addressing the determinants of PFW in ECE settings.

**Hypothesis** **H2b.** *There are significant differences in PFW between boys and girls, with boys expected to leave a slightly larger share of food uneaten than girls*.

**H2c.**  *Interaction between age and gender*

To our knowledge, no previous study has examined how age and gender jointly influence PFW in preschool settings. This exploratory hypothesis addresses this gap, proposing that the impact of age on PFW may differ between boys and girls, reflecting developmental, behavioral, and socio-cultural variations in food acceptance and consumption.

**Hypothesis** **H2c.** *In kindergarten, the interaction between age and gender has a significant effect on the amount of PFW*.

These hypotheses guided the statistical analysis described in the subsequent section.

## 3. Materials and Methods

**Figure 1:**
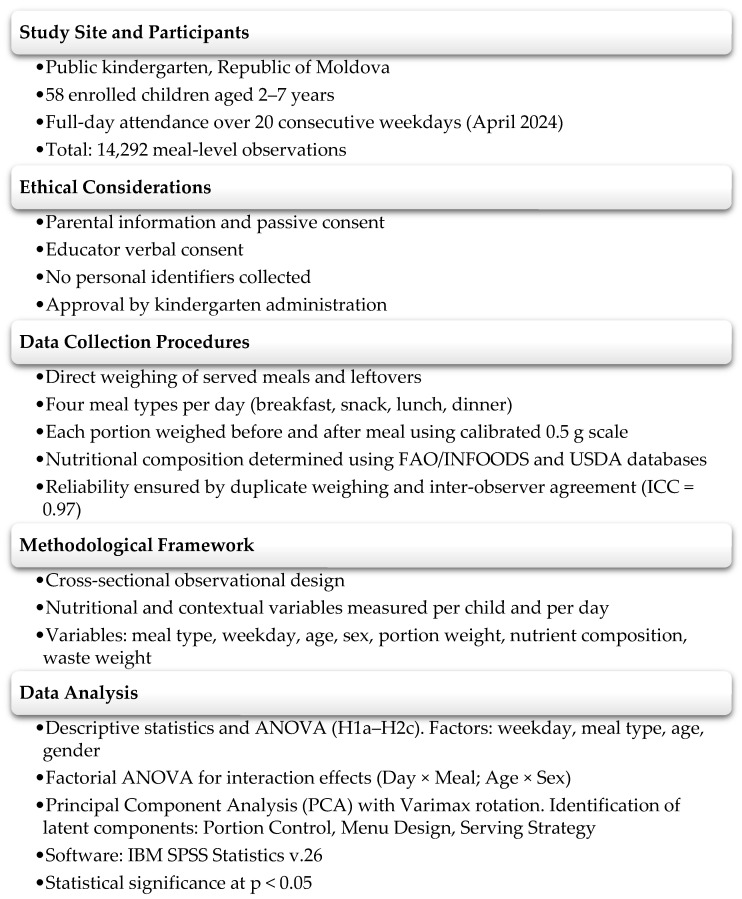
Study Design and Methodological Framework.

This research followed a cross-sectional observational design, conducted in a single kindergarten located in the Republic of Moldova, aiming to quantify PFW and identify its contextual, nutritional, and demographic determinants under serving and educational conditions (Figure 1). All statistical analyses were performed using IBM SPSS Statistics v.26, following a structured approach designed to test the research hypotheses (H1a–H2c).

The kindergarten operates on a full-day schedule of approximately 10.5 h and follows the educational policies developed by the Ministry of Education and Research of the Republic of Moldova. Staff members adhere to the national Child Development Standards for children aged 1.5 to 7 years [46], which guide daily activities and support age-appropriate learning and care. Similar institutional frameworks have been reported in other Eastern European preschool contexts [47]. The institution serves children aged 2–7 years enrolled in full-day programs, with a maximum capacity of 58 children. Participants came from diverse socio-economic backgrounds; however, demographic data were protected and are not available for publication. Breakfast, morning snack, lunch, and dinner were served daily at fixed times, with lunch lasting approximately 30 min. [48]. Between breakfast and lunch, children engaged in indoor or outdoor physical activities depending on the weather, consistent with World Health Organization (WHO) guidelines on preschool physical activity and sedentary behavior [49]. All 58 preschoolers attending the kindergarten were recruited to participate. Meals were consumed simultaneously in classrooms under identical food service conditions. Information on gender and age was obtained from institution records. Parents were informed about the study and provided passive consent, while educators granted verbal consent for children’s participation [48].

PFW was assessed through direct weighing of food served and leftovers (uneaten but still edible food remaining on children’s plates). A data sheet was designed to record, for each child and each study day, the date of data collection, the menu item offered at each eating occasion, the initial serving weight (g), and the amount of food remaining after the meal (g). Leftovers recorded three times to ensure precision, and the mean value was retained. After finishing, children were instructed to leave their plates on the table. Once all participants had completed the meal, the plates were collected. Any food scraps were scraped from the plates and weighed using a calibrated digital scale with 0.5 g precision. Food that had fallen on the table or under the chair was added to the PFW before recording [48]. Data were collected over 20 evaluation days in April 2024, covering all weekdays (Saturdays and Sundays were excluded, the kindergarten being closed), resulting in a dataset of 14,292 meal observations. During the 20 day observation period, meals were prepared according to national dietary guidelines for preschool children and served in accordance with a fixed rotation plan approved by the kindergarten. The daily menu included a balanced combination of soups, cereals, dairy products, fruits, and meat-based dishes, ensuring nutritional adequacy. The purpose of Table 1 is to describe the variables and PFW indicators used in the analysis, not to present individual menu items in full detail. The dataset included both categorical (day of the week, meal type, menu item, child age, child sex) and continuous variables (meal weight served, waste weight, protein, fat, carbohydrates, energy values served and wasted, percentage of daily nutritional requirements wasted) (Table 1).

Nutrient composition was estimated based on the FAO/INFOODS guidelines for food matching [50] and the USDA FoodData Central database [51], which provide information on the energy value and macronutrient content of foods commonly consumed in the region, ensuring comparability and reliability in dietary assessment. In line with national dietary standards, the recommended daily amounts of major food groups for children aged 1–7 years were taken from the guidelines approved by the Ministry of Health, Labour and Social Protection of the Republic of Moldova (Order No. 91/2020, in force from 1 March 2021, as amended by Order No. 62/2018) [52]. These standards specify portion sizes for cereals, fruits, vegetables, dairy products, protein-rich foods, fats, and added sugars, and were used as benchmarks for assessing plate food waste against age-appropriate serving recommendations [53]. For each food item, protein (MWP), fat (MWF), carbohydrate (MWC), and energy (MWE, kcal) values were calculated per 100 g of edible portion. These were multiplied by the actual serving weight (MealWg) and waste weight (WasteWg) to obtain: nutrients served: total protein, fat, carbohydrate, and energy in the portion; nutrients wasted (WWP, WWF, WWC, WWE): nutrients discarded with uneaten food; daily requirement loss: percentage of the minimum daily requirement (protein, fat, carbohydrate, energy) wasted, based on WHO/FAO dietary reference intakes for children aged 2–7 years. To ensure internal consistency, duplicate calculations were performed by two independent researchers. Discrepancies exceeding 1% were cross-checked and corrected. Several measures were undertaken to ensure reliability of the data: calibration (the digital scale was calibrated daily using reference weights), inter-observer reliability (two trained researchers independently weighed leftovers for a random 10% subsample; the intraclass correlation coefficient was 0.97, indicating excellent agreement); missing data handling: (observations with incomplete weight recordings (0.8% of cases) were excluded listwise from analyses).

Prior to the regression analysis, all continuous variables were assessed for compliance with statistical assumptions. Descriptive and diagnostic analyses confirmed that the data were suitable for linear modeling. The distribution of standardized residuals was approximately normal, as indicated by the absence of significant skewness or kurtosis and by the symmetrical pattern of Q–Q plots. Homoscedasticity was verified through the analysis of standardized residuals versus predicted values, showing no systematic heteroscedastic trend. The Durbin–Watson statistic (1.94–2.02 across models) confirmed the independence of residuals and absence of serial correlation. Multicollinearity was tested using Variance Inflation Factors (VIF) and tolerance coefficients. All predictors presented tolerance values above 0.10 and VIF below 10, indicating no collinearity bias in the estimated parameters. The overall model fit was highly significant, with F(6,14285) = 10,665.49, *p* < 0.001 and R^2^ = 0.818 for WasteWg, and F(6,14319) = 880.43, *p* < 0.001 and R^2^ = 0.269 for MealWg. The standardized residuals ranged between −2.31 and 1.90, confirming that no outliers exerted excessive influence on the model. Consequently, all variables satisfied the assumptions of linearity, independence, and homoscedasticity, ensuring the validity and robustness of the inferential models. Descriptive statistics (means, standard deviations, minimum, maximum) were computed for the continuous dependent variable (PFW, grams), across categorical grouping variables (weekday, meal type, age group, sex, and menu item). It is also tested the database reliability, using one-way Anova (*p*-value < 0,05) to assess differences in PFW across these groups.

To investigate the latent structure of PFW determinants, a Principal Component Analysis (PCA) with Varimax rotation was applied on 14 continuous variables related to meal weight, nutrient composition, and waste indicators (MealWg, WasteWg, MWP, MWF, MWC, MWE, WWP, WWF, WWC, WWE, FLDRP, FLDRC, FLDRL, FLDRE). Sampling adequacy was verified using the Kaiser–Meyer–Olkin (KMO) measure and Bartlett’s test of sphericity. Components with eigenvalues greater than 1 were retained, and component scores were saved for subsequent regression analysis. The PCA extracted three components explaining 84.7% of the total variance: (1) Portion Control, (2) Menu Design, (3) Serving Strategy. Together, they outline priority areas for reducing PFW and protecting the nutritional value of meals. All analyses were performed in IBM SPSS Statistics v.26. The full syntax for data processing and PCA replication is available on request.

The study was approved by the kindergarten administration and conducted in accordance with the ethical guidelines for research involving minors. No personal identifiers were recorded, and all data were anonymized prior to analysis.

## 4. Results

The empirical results are presented in two complementary stages. First, we examine the influence of temporal (weekday, meal type), nutritional (portion size, macronutrient composition, energy value), and demographic (age, sex, and their interaction) factors on PFW using univariate and factorial ANOVA models. These analyses allow us to test the specific hypotheses H1a–c and H2a–c, thereby identifying whether waste patterns vary across time, across meals, and between child subgroups. Second, we apply principal component analysis (PCA) to synthesize the multidimensional information contained in the waste and nutritional indicators into a smaller set of latent constructs. This multivariate approach provides a holistic perspective on the underlying structure of PFW, complementing the hypothesis-driven ANOVA results with data-driven evidence of common patterns and latent drivers.

### 4.1. ANOVA Results on Temporal Determinants of PFW (Day of the Week)

The one-way analysis of variance revealed statistically significant differences across weekdays, both in terms of nutrients served and nutrients discarded. With regard to nutrient provision, the amount of fat served (MWF) displayed notable variation, ranging from an average of 2.61 g on Tuesdays to 3.99 g on Thursdays, with differences confirmed by the ANOVA test (F(4,14287) = 90.190, *p* < 0.001). A similar trend was observed for energy content (MWE), where average values fluctuated between 91.7 kcal on Tuesdays and 120.9 kcal on Thursdays (F(4,14287) = 73.293, *p* < 0.001), suggesting that menu composition differed substantially across the week and potentially contributed to divergent consumption patterns among children. Equally important, the results demonstrate that nutrient losses due to PFW were not evenly distributed across weekdays. Protein waste (WWP) increased from 0.39 g on Mondays to 0.62 g on Fridays, a difference that was statistically significant (F(4,14287) = 10.629, *p* < 0.001). Fat waste (WWF) exhibited an even sharper gradient, with averages rising from 0.26 g on Mondays to 0.47 g on Fridays (F(4,14287) = 30.622, *p* < 0.001). Carbohydrate losses (WWC) followed a comparable trajectory, with daily means progressing from 1.10 g on Mondays to 1.63 g on Fridays (F(4,14287) = 12.927, *p* < 0.001). The same applied to energy waste (WWE), which increased from 8.54 kcal at the beginning of the week to 13.60 kcal at the end (F(4,14287) = 18.927, *p* < 0.001). Moreover, both serving weight (MealWg) and absolute plate waste (WasteWg) differed significantly between days of the week. While the largest portions were served at the beginning of the week (114.8 g on Monday) and on Thursday (107.8 g), the highest absolute level of waste was recorded on Friday (12.95 g), with a statistically significant difference compared to Monday (mean difference = 4.12 g, *p* < 0.001), see Table 2.

Taken together, these findings validate hypothesis H1a by demonstrating that the day of the week significantly influences children’s PFW behavior in preschool settings. The results suggest that weekly menu rotation, portion planning, and perhaps children’s varying levels of appetite and attention across the school week contribute to systematic fluctuations in nutrient intake and waste.

### 4.2. ANOVA Results on Temporal Determinants of PFW (Type of Meal Served)

The one-way ANOVA results demonstrated highly significant differences in both the nutritional composition of meals and the amount of PFW depending on the type of meal served. Average portion weights varied substantially, from 49.3 g at snacks to 124.2 g at lunch (F(3,14288) = 569.5, *p* < 0.001), while absolute PFW ranged from only 3.6 g at snacks to 13.5 g at lunch (F(3,14255) = 67.4, *p* < 0.001). These results confirm that lunch generated the largest share of PFW in absolute terms, followed by breakfast (10.8 g), dinner (8.6 g), and snacks (3.6 g). Nutrient losses reflected this same hierarchy. Protein waste (WWP) was significantly higher at lunch (0.70 g) than at breakfast (0.34 g), dinner (0.28 g), or snacks (0.04 g), with post hoc Tukey tests indicating that all pairwise differences were statistically significant (*p* < 0.001), except between breakfast and dinner. Fat waste (WWF) also peaked at lunch (0.44 g), compared to breakfast (0.31 g), dinner (0.29 g), and snacks (0.01 g). Similarly, carbohydrate losses (WWC) were greatest at lunch (1.67 g), followed by breakfast (1.39 g), dinner (0.95 g), and snacks (0.53 g). In energetic terms, wasted calories (WWE) were highest during lunch (13.8 kcal), followed by breakfast (9.9 kcal), dinner (7.7 kcal), and snacks (2.4 kcal), all differences being statistically significant (F(3,14288) = 100.6, *p* < 0.001), see Table 3.

The robust tests of equality of means (Welch, Brown-Forsythe) confirmed the stability of these results, indicating that the effects are not artifacts of variance heterogeneity. Collectively, the evidence strongly validates hypothesis H1b, showing that PFW in preschool settings is not evenly distributed across meals. Lunch is the critical period of PFW generation, accounting for the highest amounts of discarded food and nutrients, while snacks contribute only marginally to total losses.

### 4.3. Factorial ANOVA Results on Temporal Determinants of PFW (Day of the Week * Type of Meal Served)

The factorial ANOVA revealed a statistically significant interaction between weekday and meal type, indicating that the effect of weekday on portion weight is not consistent across all meals. Specifically, the interaction term Day × Meal was significant (F(12,14272) = 14.82, *p* < 0.001, partial η^2^ = 0.012). This result demonstrates that portion sizes and by extension, the potential for PFW, are shaped simultaneously by temporal (weekday) and structural (meal type) factors. The descriptive statistics confirm that lunch consistently represented the largest portions across all weekdays, ranging from 122.0 g on Wednesdays to 128.1 g on Fridays, while snacks remained the smallest, averaging around 49 g irrespective of the day. However, the interplay between weekdays and meals was evident for breakfast and dinner, where mean serving weights fluctuated substantially: breakfast portions were larger at the beginning of the week (127.6 g on Monday) and declined towards the end (101.2 g on Friday), while dinner portions showed a similar drop, from 107.9 g on Monday to only 85.3 g on Friday. These variations suggest that institutional planning and weekly menu cycles introduce systematic changes in portion allocation, with implications for both nutrient intake and waste generation. The main effects of the independent variables also proved significant. The effect of meal type was particularly strong (F(3,14272) = 570.44, *p* < 0.001, partial η^2^ = 0.107), confirming that meal category is the most important determinant of portion weight. The main effect of weekday was also significant though smaller in magnitude (F(4,14272) = 13.64, *p* < 0.001, partial η^2^ = 0.004), reflecting moderate variation across the week. Together, the corrected model explained 12.1% of the variance in portion weights (adjusted R^2^ = 0.120).

The univariate model, which incorporated meal weight, macronutrient composition, energy values, nutrient-specific waste, and percentages of daily requirement losses as covariates, confirmed the robustness of the effects of both weekday and meal type, as well as their interaction, on PFW (WasteWg). The overall corrected model explained a substantial share of the variance (F(20,14238) = 112.38, *p* < 0.001), with an adjusted R^2^ of 0.12, indicating that approximately 12% of the variability in waste could be attributed to the combined influence of temporal, structural, and nutritional predictors. The effect of the intercept remained highly significant (F(1,14238) = 1362.51, *p* < 0.001), underscoring the central role of baseline consumption and PFW patterns in the kindergarten setting. Critically, the interaction between weekday and meal type retained statistical significance after adjusting for the nutritional covariates (F(12,14238) = 23.12, *p* < 0.001, partial η^2^ = 0.012). This demonstrates that the observed fluctuations in waste are not solely the result of differences in serving size or nutrient density but also reflect the interplay between when (weekday) and what (meal type) children are served. Lunch consistently generated the largest quantities of waste across weekdays (mean range: 12.6–17.0 g), while snacks remained the least wasteful (means between 1.0 and 6.1 g), with breakfast and dinner showing greater variability across days. For example, breakfast waste peaked on Wednesdays (14.4 g) but fell to 6.7 g on Thursdays, while dinner waste varied from 15.9 g on Thursdays to less than 1 g on Fridays, suggesting that weekly menu cycles and portioning practices introduce structural variability that goes beyond nutritional composition. The main effect of weekday was significant though of modest magnitude (F(4,14238) = 8.90, *p* < 0.001, partial η^2^ = 0.004), indicating that the day of the week independently influences PFW. In contrast, the main effect of meal type was much stronger (F(3,14238) = 63.14, *p* < 0.001, partial η^2^ = 0.018), reflecting the structural differences between breakfast, snacks, lunch, and dinner as drivers of waste (Table 4). Importantly, the block of covariates representing portion size, macronutrient composition, and nutrient losses explained the largest share of variance (F(1,14238) = 1683.94, *p* < 0.001, partial η^2^ = 0.106), confirming that PFW is systematically linked to what and how much children are served. Robustness checks further supported these findings. Levene’s test and the Breusch–Pagan test indicated heteroskedasticity, consistent with the high variability typical of portion-level data in early childhood contexts. Nevertheless, the highly significant F-ratios across main and interaction effects reinforce the validity of the results.

Taken together, these outcomes provide strong support for H1c, demonstrating that the effect of weekday on PFW is contingent on the type of meal served, even after accounting for differences in portion size, nutrient content, and energy values. The findings underline the need to consider not only nutritional adequacy but also temporal and operational patterns when designing interventions aimed at reducing PFW in ECE settings.

**Figure 2:**
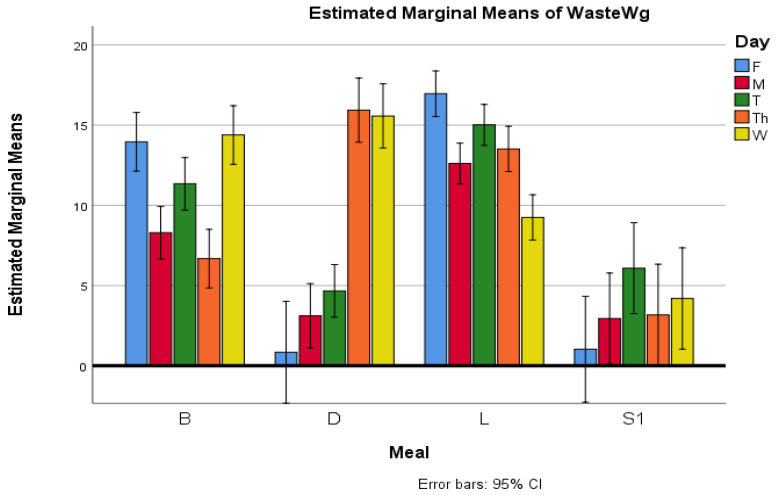
Interaction effects of meal type and weekday on PFW (WasteWg) in kindergarten. Error bars: 95% CI.

Figure 2 illustrates the interaction between weekday and meal type on PFW (WasteWg).

The profile plot shows that lunch consistently generates the highest levels of waste across weekdays, whereas snacks produce the lowest amounts. Breakfast and dinner display greater variability, with waste peaking midweek and declining towards Friday. Error bars represent 95% confidence intervals.

### 4.4. ANOVA Results on Demographic Determinants of PFW (Consumer Gender)

The results of the one-way ANOVA indicated that gender differences in PFW are relatively modest, yet statistically detectable for several nutritional indicators. Descriptive statistics showed that boys generated slightly higher mean levels of waste across most categories, with WasteWg averaging 11.37 g compared to 10.63 g for girls. Although the overall difference in absolute PFW did not reach conventional significance levels in the basic ANOVA model (F(1,14257) = 3.06, *p* = 0.080), the test of homogeneity of variances suggested heteroskedasticity (Levene’s test: F = 11.18, *p* = 0.001), which supports the robustness of examining gender-specific tendencies through complementary analyses.

At the level of nutrient-specific waste, some dimensions reached statistical significance. Boys discarded on average 0.35 g of fat per meal compared to 0.33 g for girls, with this difference confirmed by Levene’s test (F = 7.51, *p* = 0.006) and robust tests (Welch and Brown-Forsythe, both *p* < 0.05). Carbohydrate waste also differed significantly (1.40 g vs. 1.32 g), as did energy waste (10.93 kcal vs. 10.31 kcal), with homogeneity tests again indicating systematic variance differences between groups. Even more pronounced differences were observed in relative terms, with boys losing a greater proportion of their daily macronutrient requirements through PFW: proteins (WDRP, 0.029 vs. 0.026), carbohydrates (WDRC, 0.0023 vs. 0.0022), lipids (WDRL, 0.038 vs. 0.035), and energy (WDRE, 0.0092 vs. 0.0085). All these indicators were significant in at least one of the robust equality-of-means tests (*p* < 0.05), suggesting that while the absolute differences may appear small, their consistency across nutrient categories reinforces their substantive relevance.

By contrast, no significant gender differences emerged in the portion size served (MealWg), which averaged 110.23 g for both boys and girls (F(1,14290) = 0.00, *p* = 0.992). This indicates that observed differences in waste are not attributable to differences in the amount of food offered, but rather to behavioral and preference-related factors (Table 5).

These results partially confirm hypothesis H2b. Boys in kindergarten tend to leave a slightly larger share of food uneaten compared to girls, particularly in terms of nutrient-dense items such as fats and carbohydrates, as well as relative losses of daily nutritional requirements. Although effect sizes are small, the consistent pattern across multiple indicators suggests that gender is a relevant explanatory variable in studies of PFW in early childhood education settings.

### 4.5. ANOVA Results on Demographic Determinants of PFW (Consumer Age)

The analysis does not support the hypothesis that PFW indicators vary significantly across age categories. Rather, the results suggest that age-related differences are negligible and that the distribution of environmental performance measures is consistent across demographic groups. Consequently, Hypothesis 2 is not validated in the case of age, in contrast to weekday-related differences observed previously, indicating that behavioral or structural determinants other than age may be more relevant in explaining variability in PFW outcomes (Table 6).

### 4.6. Factorial ANOVA Results on Demographic Determinants of PFW (Consumer Gender * Consumer Age)

The factorial ANOVA assessing the effects of age, sex, and their interaction on portion size (MealWg) in kindergarten settings did not reveal any meaningful differences. The corrected model explained virtually none of the variance in portion weights (R^2^ ≈ 0.000, adjusted R^2^ < 0). The main effect of age was nonsignificant (F(5,14280) = 0.001, *p* = 1.000), as was the effect of sex (F(1,14280) = 0.000, *p* = 1.000). Most importantly, the Age × Sex interaction also failed to reach significance (F(5,14280) = 0.001, *p* = 1.000), indicating that portion sizes were distributed uniformly across demographic subgroups. The factorial ANOVA, adjusted for portion size, nutrient composition, and nutrient waste covariates, revealed that the interaction between age and sex has a statistically significant effect on PFW in kindergarten. The Age × Sex term was significant (F(5,14246) = 8.08, *p* < 0.001, partial η^2^ ≈ 0.005), indicating that the relationship between age and PFW differs between boys and girls. While neither age (F(5,14246) = 0.83, *p* = 0.531) nor sex (F(1,14246) = 1.65, *p* = 0.199) alone explained significant variation in waste, their combined effect was robust, demonstrating that demographic influences on PFW are contingent rather than additive. The descriptive statistics illustrate this pattern clearly. Among three- and four-year-olds, boys produced substantially more waste than girls (13.0 g vs. 9.5 g at age 3; 13.4 g vs. 9.1 g at age 4), suggesting that early developmental differences in eating behavior and food acceptance are more pronounced in boys. Conversely, at age six, the pattern reversed, with girls wasting more (11.9 g) compared to boys (9.0 g). At age seven, the gender gap narrowed again (13.0 g vs. 10.8 g). These fluctuations demonstrate that PFW trajectories across childhood are not linear but interact with gendered developmental pathways, including autonomy in food choices, neophobia, and socialization patterns. The covariate block (MealWg, MWP, MWF, MWC, MWE, WWP, WWF, WWC, WWE, FLDRP, FLDRC, FLDRL, FLDRE) accounted for the majority of variance (F(1,14246) = 1692.79, *p* < 0.001, partial η^2^ ≈ 0.106), confirming that the nutritional context remains the primary determinant of waste. However, the additional explanatory power of the Age × Sex interaction emphasizes the necessity of considering demographic heterogeneity in interventions. Robustness tests (Levene’s and Breusch–Pagan) indicated significant heteroskedasticity (*p* < 0.001), which is expected in portion-level observational data, but the effect sizes and consistency across age groups validate the interaction (Table 7).

**Figure 3:**
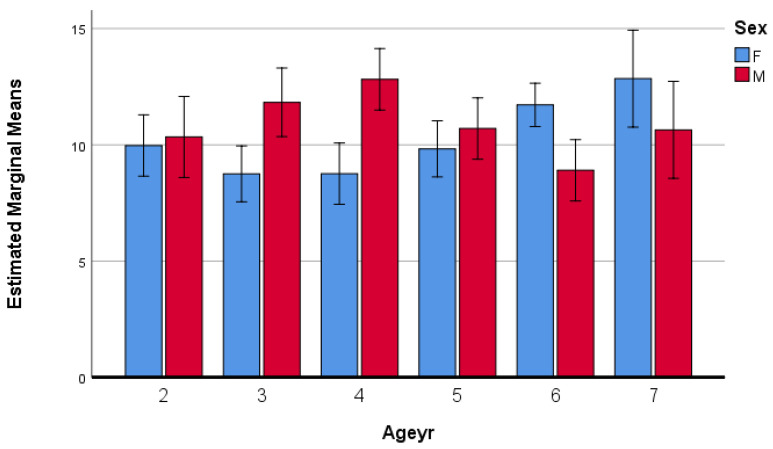
Interaction effects of age and sex on PFW (WasteWg) in kindergarten. Error bars: 95% CI.

Figure 3 illustrates the interaction between age and sex on PFW (WasteWg). The profile plot shows that boys generate higher levels of waste at ages 3–4, whereas girls surpass boys at ages 5–6, with the gap narrowing again at age 7. Error bars represent 95% confidence intervals.

Together, these findings support hypothesis H2c, highlighting that the influence of age on PFW cannot be generalized across sexes. Instead, boys and girls follow distinct developmental trajectories in food acceptance and waste behavior, with implications for tailoring nutritional education and menu planning in preschool institutions.

### 4.7. Multivariate Patterns of PFW (PCA Components)

To complement the hypothesis-driven ANOVA models, a principal component analysis (PCA) was conducted with the aim of synthesizing the multidimensional information contained in the set of waste and nutritional indicators into a smaller number of latent constructs. The objectives of this analysis were threefold: (i) to reduce data dimensionality while preserving as much variance as possible, (ii) to identify clusters of highly correlated variables that reflect underlying drivers of PFW, and (iii) to provide an integrative framework for interpreting the joint influence of temporal, nutritional, and demographic factors. By applying PCA, the study moves beyond isolated hypothesis testing and explores the structural relationships among the key indicators of PFW, including portion weight (MealWg), absolute and relative measures of waste (WasteWg, WWP, WWF, WWC, WWE), and nutrient-specific losses in relation to daily requirements (FLDRP, FLDRC, FLDRL, FLDRE). The latent components extracted through PCA allow for a more holistic understanding of how portioning practices, nutrient composition, and requirement-adjusted inefficiencies interact to form coherent patterns of waste in kindergarten settings.

The communalities analysis (Table 8) provide the basis for assessing the adequacy of the variables included in the principal component analysis. Initial communalities are all equal to 1.000, as expected in PCA extraction, while extraction communalities vary between 0.62 and 0.83 across indicators. This range indicates that between 62% and 83% of the variance of each variable is accounted for by the retained components.

**Figure 4:**
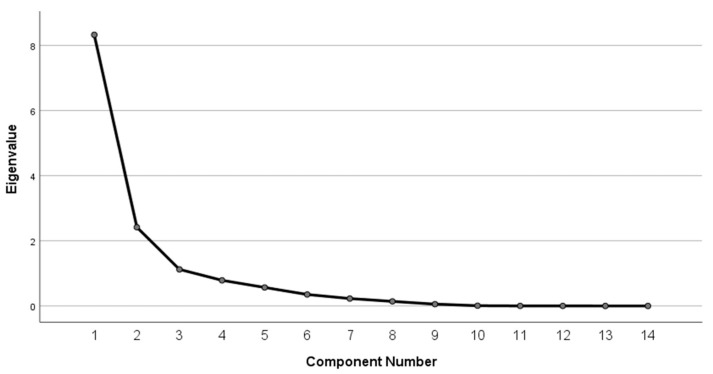
Scree plot.

The eigenvalue analysis and scree plot (Figure 3) confirm the adequacy of a three-component solution. The first component has an eigenvalue of 8.33, explaining 59.48% of the total variance. The second component adds 2.42 eigenvalue units, contributing 17.28% of the variance, while the third component retains 1.12 eigenvalue units, explaining 8.01%. Together, these three components account for 84.77% of the cumulative variance, a very high proportion that indicates an efficient dimensionality reduction (Figure 4).

The scree plot displays the characteristic “elbow” after the third component, where the slope of the curve sharply decreases and subsequent components contribute marginally (all with eigenvalues < 1.0, each explaining less than 6% of the variance). According to Kaiser’s criterion (eigenvalues greater than 1.0) and Cattell’s scree test, only the first three components should be retained for interpretation, as they capture the substantive variance and structure in the dataset.

**Figure 5:**
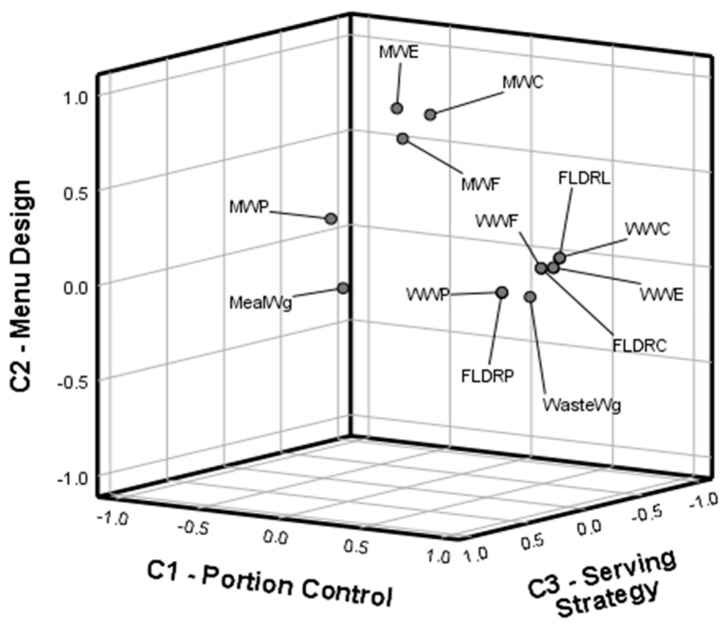
Component plot in rotated space.

The rotated component matrix and Component plot in rotated space (Figure 5), provides strong numerical evidence for a three-dimensional structure of PFW, which can be causally interpreted as Portion Control, Menu Design, and Serving Strategy.

The first component, Portion Control, displays very high loadings, such as 0.937 for FLDRE, 0.937 for WWE, 0.932 for WWC, 0.929 for FLDRL, and 0.917 for FLDRC. This statistical clustering indicates that when portion sizes are not carefully managed, waste is not only larger in grams but also nutritionally costly, with energy and macronutrients systematically lost. In more practical terms, this means that excess food placed on children’s plates translates directly into calories, carbohydrates, and proteins that never get consumed. The causal interpretation is clear: weak portion control drives inefficiency, both in absolute and nutritional terms, reinforcing the need for serving amounts tailored to children’s real consumption capacity.

The second component, Menu Design, is represented by high loadings for meal composition variables: MWE (0.918), MWC (0.888), and MWF (0.771). This pattern shows that the nutritional profile of meals—the energy density, the carbohydrate balance, and the fat content—is itself a latent driver of waste. Meals with higher caloric density or a heavier balance of certain macronutrients are more likely to generate leftovers, not because children are served too much food, but because the design of the menu influences acceptance. For example, dishes richer in carbohydrates or fat may be less appealing or harder to finish, thus indirectly shaping waste patterns. From a causal perspective, how meals are composed is as critical as how much is served, because children’s preferences and satiety cues interact with the nutritional structure of the food.

The third component, Serving Strategy, has its highest loadings on MealWg (0.722) and MWP (0.789). This suggests that the way portions are constructed, particularly the balance between total weight and protein content, forms a separate dimension of waste. In practice, this reflects institutional serving norms: when meals are distributed with a high protein load relative to portion size, children may not consume the entire serving, leading to selective waste. Thus, serving strategy operates independently from overall waste intensity and from nutritional density, pointing to the role of pedagogical and operational choices in how meals are offered.

Variables cluster into distinct groups: waste and requirement-adjusted indicators align strongly with Portion Control, nutritional composition indicators align with Menu Design, while portion size and protein indicators align with Serving Strategy. This separation demonstrates that PFW is not the product of a single cause but emerges from three distinct but interacting processes: too much food served, menus that are not fully aligned with children’s preferences, and serving norms that do not match children’s eating behaviors.

## 5. Discussion

This study aimed to identify the determinants of PFW in ECE and to explore their underlying structure by combining hypothesis-driven variance analysis with PCA. The working hypotheses (H1a–c, H2a–c) addressed temporal, nutritional, and demographic effects of PFW, while the multivariate analysis aimed to capture broader latent constructs that integrate these influences. The results provide important insights into both the specific conditions under which waste arises and the structural patterns that sustain it.

The ANOVA models confirmed that temporal variation is a consistent determinant of PFW. Waste levels differed significantly across weekdays and meal types, with lunch generating the largest quantities of waste and snacks the lowest. Breakfast and dinner showed more variability, with midweek peaks and declines toward Friday, reflecting menu cycles and attendance dynamics. These findings are consistent with earlier research that emphasizes the role of temporal and contextual factors in shaping children’s eating behavior [36,40,41,48]. Hypothesis H1a and H1b were therefore validated, and the significant interaction between weekday and meal type (H1c) highlighted that these factors exert their effects in combination rather than isolation.

Demographic analyses yielded a more nuanced picture. Although age alone did not explain significant differences in waste, gender analyses indicated that boys left slightly more food uneaten, particularly for nutrient-dense items such as fats and carbohydrates. This partial confirmation of H2b resonates with studies suggesting that boys’ eating patterns are more inconsistent and selective [43,44]. Moreover, the factorial model revealed that the effect of age is contingent on gender, with boys producing more waste at ages three and four, while girls surpassed boys at ages five and six, before converging again at age seven. Evidence from Republic of Moldova supports this demographic heterogeneity, showing that parental socio-demographic characteristics significantly shape children’s dietary patterns [54]. This interaction (H2c) is particularly important, as it demonstrates that developmental trajectories cannot be generalized across sexes. Similar crossovers have been noted in primary school research, where socialization, autonomy in food choices, and neophobia interact to shape food acceptance [38,39,42]. The present study therefore adds to the literature by showing that such demographic heterogeneity is already visible at the kindergarten stage.

A key contribution of this research lies in moving beyond univariate associations through PCA. The three retained components explained more than 84% of the variance and offered a parsimonious framework for interpreting PFW. The first component, Portion Control, brought together absolute and requirement-adjusted indicators of waste, with loadings as high as 0.937 for energy waste (WWE) and 0.932 for carbohydrate waste (WWC). This indicates that excessive servings directly translate into nutrient and energy losses, supporting the causal interpretation that inadequate portion management is the dominant driver of waste [55]. Previous studies confirm that portion size exerts a strong influence on children’s intake and PFW, with smaller initial servings and flexible second helpings shown to reduce waste without compromising nutritional adequacy [55,56]. The second component, Menu Design, was defined by energy and macronutrient composition variables (MWE = 0.918, MWC = 0.888, MWF = 0.771), showing that the nutritional profile of meals influences acceptance independently of serving size. This highlights that waste is not only about “how much” is served but also about “what” is served, and how sensory attributes such as texture affect children’s willingness to eat [18,57,58,59]. The third component, Serving Strategy, captured the relationship between portion size and protein content (MealWg = 0.722, MWP = 0.789), pointing to institutional practices in food distribution. Taken together, these components provide an integrated understanding of PFW, consistent with the threefold objectives of the analysis: dimensionality reduction, cluster identification, and integrative framework building. From a theoretical perspective, the study contributes to the literature by showing that PFW in early childhood settings cannot be explained by isolated factors but must be understood as the outcome of interacting structural, nutritional, and behavioral determinants. This multidimensional approach advances previous work, which has often focused on single drivers, by situating PFW within a coherent set of latent constructs. Systematic reviews confirm that waste patterns in school settings are complex and shaped by overlapping drivers, supporting the use of integrated frameworks like PCA [60]. Comparable studies in early education settings have reported similar levels of PFW. For instance, Kasavan et al. [20] found that 17.4% of total food prepared in Malaysian school canteens became waste, Filimonau et al. [21] observed that most waste in Russian kindergartens occurred on children’s plates, and Nguyen et al. [22] reported an average of 23% of food served being discarded in Vietnamese primary schools. These findings suggest that FW in early education contexts is largely influenced by portion control, menu management, and sensory appeal rather than by national income level. The novelty of the findings lies particularly in the Eastern European context, where systematic studies of kindergarten FW remain scarce.

From a policy perspective, the findings underscore the need for institutional guidelines that establish sustainable standards for meal provision in ECE. Current practices in many kindergartens remain guided by rigid nutritional norms and cost-efficiency concerns, often overlooking the interplay between portion size, menu composition, and developmental needs. The evidence here suggests that integrated frameworks should address three complementary dimensions: portion control, menu design, and serving strategy. Smaller initial portions with the option of second helpings could reduce requirement-adjusted waste while safeguarding nutritional adequacy, consistent with international experiences [55,56]. Menu design should be treated as a policy tool rather than an operational detail, with guidelines considering acceptability, cultural relevance, and sensory appeal [58]. Serving strategy also requires explicit recognition, encouraging practices such as modular plating, pedagogical involvement, and responsive feeding strategies. Reviews confirm the effectiveness of such interventions in reducing institutional FW and supporting behavioral change [61,62].

Integrating these dimensions into policy would extend beyond immediate waste reduction. It would promote nutritional equity by ensuring acceptable meals across socio-economic groups [63], reduce environmental impact in line with sustainability goals [5,64], and support public health by fostering healthy eating trajectories early in life. Evidence from Italy shows that children’s PFW carries not only nutritional but also environmental and economic implications, reinforcing its systemic nature [65]. Recent assessments in Republic of Moldova also underline the importance of aligning nutritional security policies with international standards, confirming that institutional practices are part of a broader governance framework [66].

Finally, the implications of reducing PFW extend beyond the boundaries of ECE and nutrition, linking directly to global food security and international stability. Increasingly, FW reduction is recognized as a strategic tool for mitigating humanitarian crises and preventing geopolitical instability, as recent disruptions to agricultural supply chains have underscored the urgent need for coordinated international responses [67].

Global FAO assessments emphasize that reducing FW is essential for achieving Sustainable Development Goal 12.3 and can only be realized through strong multilateral cooperation [64]. Integrating preschool PFW data into transnational monitoring systems could strengthen such frameworks. Platforms like HungerMapLIVE [68] and the proposed harmonized HFID dataset [69] show how localized data can feed into predictive analytics, enabling early detection of vulnerabilities and better resource allocation [70]. Taken together, these insights show that reducing PFW in ECE is not a marginal concern but part of a global agenda of food security and resilience.

This exploratory study presents several strengths, including the direct and systematic quantification of PFW, the inclusion of all children enrolled in the institution (n = 58), and the collection of 14,292 meal-level observations under consistent nutritional and operational conditions. The close involvement of the kindergarten management also ensured data accuracy and compliance with daily procedures. However, the study’s single-site design and limited sample size restrict the generalizability of findings to other educational contexts. Future research should expand to multiple kindergartens across different regions to validate and extend the patterns observed in this pilot investigation.

## 6. Conclusions

This study shows that PFW in ECE is driven by a combination of temporal, nutritional, and demographic factors. Lunch emerged as the main contributor to waste, while gender- and age-related interactions highlighted heterogeneous developmental trajectories. The principal component analysis reduced the complexity of waste patterns to three key dimensions: portion control, menu design, and serving strategy, demonstrating that PFW cannot be attributed to a single factor but to the interplay between serving practices, nutritional composition, and children’s behavioral responses. These insights underscore the need for institutional guidelines that go beyond rigid nutritional standards. Policies promoting smaller initial portions with flexible second helpings, nutritionally adequate but sensorially appealing menus, and responsive serving strategies can reduce waste while supporting equity, sustainability, and healthier eating habits. Moreover, integrating preschool PFW data into global monitoring frameworks would align local interventions with international agendas such as SDG 12.3, positioning ECE as both a driver of behavioral change and a contributor to resilient food systems. Nevertheless, the study’s single-site, small-sample design represents a limitation that restricts the generalizability of the findings. Future research should expand to multiple kindergartens and diverse regional contexts to confirm the observed patterns and develop broader strategies for reducing PFW in early education institutions. Potential measurement errors were minimized by using calibrated electronic kitchen scales with ±1 g precision and standardized weighing procedures. Macronutrient composition was derived from national food composition tables corresponding to each menu item rather than direct chemical analysis. Although small deviations may occur due to variation in cooking and portion size, the use of consistent recipes and uniform weighing protocols across all observation days ensured reliable and comparable data. In addition to structural and nutritional factors, children’s sensory preferences and behavioral responses also contribute to PFW in early education settings. Taste acceptance, familiarity with specific foods, and appetite fluctuations can strongly influence the proportion of uneaten meals. Several studies have reported that children are more likely to waste foods perceived as less palatable, especially mixed dishes or those with complex textures. These behavioral aspects underline the importance of designing menus that are not only nutritionally balanced but also aligned with children’s sensory preferences and gradual taste development. Future research should extend this work through multi-site and longitudinal designs covering diverse educational and cultural contexts. Studies integrating direct observation with sensory evaluation and behavioral interventions would help disentangle the combined effects of taste preference, portion control, and menu structure. Furthermore, experimental trials introducing educational activities or participatory menu planning could provide practical insights into how awareness and engagement influence PFW reduction among preschool children.

## Figures and Tables

**Table 1:** Description of variables used in the statistical analysis, including symbols, variable names, and details of measurement scales and nutritional parameters related to meal composition and PFW indicators.

Variable Symbol	Variable Name	Variable Details (Scale)											
Day	Observation day	M—Monday		T—Tuesday			W—Wednesday		Th—Thursday			F—Friday	
Meal	Meal type	B-Breakfast			S1-Snack 1			L—Lunch			D-Dinner		
Ageyr	Child’s age (years)	2	3			4		5		6			7
Sex	Child sex	M						F					
Menu	Menu item	CM—Cereal with milk; BB—Bread with butter; HT—Herbal tea; BM—Borscht with meat; BV—Borscht with vegetables; MD—Meat with side dish; SV—Salads and vegetables; BD—Bread; FC—Fruit compote; FR—Fruit; CD—Cheese dishes; DP—Dairy products; SW—Processed sweets; BE—Boiled eggs; BJ—Bread with jam.											
MealWg	Initial recording of meal weight, (grams)	31–222 g											
WasteWg	Average weight at the end of the meal, (grams)	0–200 g											
MWP	Weight of the meal served—Protein, (grams)	CM—3.5 *; BB—7 *; HT—0 *; SM—3.9 *; SV—3.85 *; MD—11 *; SV—1 *; BD—9 *; FC—0.5 *; FR—1 *; CD—9 *; DP—3.5 *; SW—6 *; BE—13 *; BJ—6 *.											
MWF	Weight of the meal served—Fat, (grams)	CM—2 *; BB—17.5 *; HT—0 *; SM—3.5 *; SV—3.8 *; MD—3 *; SV—5 *; BD—2 *; FC—0 *; FR—0.3 *; CD—8 *; DP—3.5 *; SW—25 *; BE—11 *; BJ—3 *.											
MWC	Weight of the meal served—Carbohydrates, (grams)	CM—14 *; BB—40 *; HT—0 *; SM—10 *; SV—12 *; MD—9 *; SV—3 *; BD—52 *; FC—20 *; FR—15 *; CD—14 *; DP—4.5 *; SW—65 *; BE—1 *; BJ—60 *.											
MWE	Energy value of meal, (kcal.)	CM—90.35 **; BB—355.45 **; HT—0 **; SM—89.54 **; SV—100.325 **; MD—109.9 **; SV—62.9 **; BD—268.7 **; FC—84.05 **; FR—68.39 **; CD—168.7 **; DP—65.35 **; SW—523.6 **; BE—159.7 **; BJ—298.5 **.											
WWP	Waste weight of the meal served—Protein, (grams)	CM—3.5 *; BB—7 *; HT—0 *; SM—3.9 *; SV—3.85 *; MD—11 *; SV—1 *; BD—9 *; FC—0.5 *; FR—1 *; CD—9 *; DP—3.5 *; SW—6 *; BE—13 *; BJ—6 *.											
WWF	Waste weight of the meal served—Fat, (grams)	CM—2 *; BB—17.5 *; HT—0 *; SM—3.5 *; SV—3.8 *; MD—3 *; SV—5 *; BD—2 *; FC—0 *; FR—0.3 *; CD—8 *; DP—3.5 *; SW—25 *; BE—11 *; BJ—3 *.											
WWC	Waste weight of the meal served—Carbohydrates, (grams)	CM—14 *; BB—40 *; HT—0 *; SM—10 *; SV—12 *; MD—9 *; SV—3 *; BD—52 *; FC—20 *; FR—15 *; CD—14 *; DP—4.5 *; SW—65 *; BE—1 *; BJ—60 *.											
WWE	Waste Energy value of meal, (kcal.)	CM—90.35 **; BB—355.45 **; HT—0 **; SM—89.54 **; SV—100.325 **; MD—109.9 **; SV—62.9 **; BD—268.7 **; FC—84.05 **; FR—68.39 **; CD—168.7 **; DP—65.35 **; SW—523.6 **; BE—159.7 **; BJ—298.5 **.											
FLDRP	Food losses % minimum daily protein requirement	minimum daily protein requirement 2-year-old child 13 g; 3-year-old child 13 g; 4-year-old child 16 g; 5-year-old child 19 g; 6-year-old child 22 g; 7-year-old child 24 g.											
FLDRC	Food losses % minimum daily carbohydrates requirement	minimum daily carbohydrates requirement 2-year-old child 130 g; 3-year-old child 130 g; 4-year-old child 150 g; 5-year-old child 160 g; 6-year-old child 170 g; 7-year-old child 180 g.											
FLDRL	Food losses % minimum daily lipids requirement	minimum daily lipids requirement 2-year-old child 30 g; 3-year-old child 30 g; 4-year-old child 35 g; 5-year-old child 45 g; 6-year-old child 45 g; 7-year-old child 50 g.											
FLDRE	Food losses % Min. energy value requirement	minimum energy value requirement 2-year-old child 1000 kcal; 3-year-old child 1000 kcal; 4-year-old child 1200 kcal; 5-year-old child 1300 kcal; 6-year-old child 1400 kcal; 7-year-old child 1500 kcal.											

Legend: CM—Cereal with milk; BB—Bread with butter; HT—Herbal tea; BM—Borscht with meat; BV—Borscht with vegetables; MD—Meat with side dish; SV—Salads and vegetables; BD—Bread; FC—Fruit compote; FR—Fruit; CD—Cheese dishes; DP—Dairy products; SW—Processed sweets; BE—Boiled eggs; BJ—Bread with jam; * %/100 g of food; ** Kcal/100 g of food.

**Table 2:** ANOVA results and descriptive statistics of plate food waste indicators by weekday (Mean ± SD).

Weekday	WasteWg (g)	WWP (g)	WWF (g)	WWC (g)	WWE (kcal)
Monday	8.83 ± 18.88	0.39 ± 1.21	0.26 ± 0.60	1.11 ± 2.32	8.54 ± 18.16
Tuesday	10.75 ± 27.54	0.43 ± 1.39	0.30 ± 0.92	1.37 ± 3.48	10.22 ± 26.68
Wednesday	11.52 ± 21.96	0.43 ± 0.94	0.28 ± 0.65	1.20 ± 2.50	9.29 ± 18.57
Thursday	11.36 ± 26.38	0.48 ± 1.58	0.42 ± 1.07	1.51 ± 3.38	12.07 ± 27.30
Friday	12.95 ± 29.54	0.62 ± 1.60	0.47 ± 1.07	1.63 ± 3.70	13.60 ± 29.97
F(df = 4,14287)	10	10.629	30.622	12.927	18.927
*p*-value *	<0.001	<0.001	<0.001	<0.001	<0.001

* All tests significant at the 0.001 level.

**Table 3:** ANOVA results and descriptive statistics of PFW indicators by meal type (Mean ± SD).

Meal Type	WasteWg (g)	WWP (g)	WWF (g)	WWC (g)	WWE (kcal)	Portion (MealWg, g)
Breakfast	10.83 ± 23.0	0.34 ± 0.77	0.31 ± 0.72	1.39 ± 3.28	9.93 ± 22.1	112.80 ± 62.00
Snack	3.61 ± 7.30	0.04 ± 0.07	0.01 ± 0.02	0.53 ± 1.09	2.44 ± 5.00	49.30 ± 4.00
Lunch	13.49 ± 29.60	0.70 ± 1.86	0.44 ± 1.06	1.67 ± 3.47	13.83 ± 29.80	124.20 ± 67.90
Dinner	8.57 ± 20.00	0.28 ± 0.63	0.29 ± 0.78	0.95 ± 2.45	7.72 ± 17.10	102.80 ± 51.00
F(df = 3,14288)	67.41	139.86	94.61	68.87	100.58	569.51
*p*-value *	<0.001	<0.001	<0.001	<0.001	<0.001	<0.001

* All tests significant at the 0.001 level.

**Table 4:** Two-way ANOVA results and descriptive statistics of PFW by weekday and meal type (Mean ± SD, g).

MealWg	Breakfast	Snack	Lunch	Dinner	Total
Monday	127.6 ± 62.6	50.0 ± 4.6	123.0 ± 65.0	107.9 ± 39.2	114.8 ± 61.1
Tuesday	109.0 ± 63.5	46.0 ± 3.4	125.2 ± 68.3	93.0 ± 61.9	106.5 ± 66.4
Wednesday	124.6 ± 58.8	49.3 ± 2.6	122.0 ± 67.2	111.5 ± 59.4	114.1 ± 63.7
Thursday	98.8 ± 59.1	53.0 ± 3.0	122.9 ± 70.1	110.6 ± 37.8	107.8 ± 61.1
Friday	101.2 ± 59.4	49.0 ± 1.2	128.1 ± 69.5	85.3 ± 14.3	107.9 ± 64.1
Total	112.8 ± 62.0	49.3 ± 4.0	124.2 ± 67.9	102.8 ± 51.0	110.2 ± 63.5
**WasteWg**	**Breakfast**	**Snack**	**Lunch**	**Dinner**	**Total**
Monday	8.30 ± 17.26	2.94 ± 6.53	12.61 ± 22.80	3.11 ± 10.40	8.83 ± 18.88
Tuesday	11.35 ± 26.28	6.08 ± 8.63	15.01 ± 36.07	4.66 ± 9.30	10.75 ± 27.54
Wednesday	14.39 ± 25.07	4.19 ± 9.18	9.25 ± 19.69	15.57 ± 24.46	11.52 ± 21.96
Thursday	6.68 ± 15.56	3.16 ± 6.18	13.52 ± 30.58	15.94 ± 30.72	11.36 ± 26.38
Friday	13.96 ± 27.59	1.03 ± 3.07	16.96 ± 34.65	0.84 ± 2.52	12.95 ± 29.54
Total	8.30 ± 17.26	2.94 ± 6.53	12.61 ± 22.80	3.11 ± 10.40	8.83 ± 18.88
**Two-way ANOVA MealWg**	**F (df)**			*p*-value *	**Partial η^2^**
Day (weekday)	F(4,14272) = 13.64			<0.001	0.004
Meal (meal type)	F(3,14272) = 570.44			<0.001	0.107
Day × Meal	F(12,14272) = 14.82			<0.001	0.012
**Two-way ANOVA WasteWg**	**F (df)**			*p*-value *	**Partial η^2^**
Day (weekday)	F(4,14238) = 8.90			<0.001	0.004
Meal (meal type)	F(3,14238) = 63.14			<0.001	0.018
Day × Meal	F(12,14238) = 23.12			<0.001	0.012
Covariates block	F(1,14238) = 1683.94			<0.001	0.106

* All tests significant at the 0.001 level.

**Table 5:** ANOVA results and descriptive statistics of PFW indicators by gender (Mean ± SD).

Gender	WasteWg (g)	WWP (g)	WWF (g)	WWC (g)	WWE (kcal)
Boys (Mean ± SD)	11.37 ± 25.70	0.48 ± 1.36	0.35 ± 0.89	1.40 ± 3.17	10.93 ± 24.83
Girls (Mean ± SD)	10.63 ± 24.51	0.45 ± 1.36	0.33 ± 0.87	1.32 ± 3.07	10.31 ± 24.17
F(df = 1,14290)	3.06	1.38	2.06	2.1	2.25
*p*-value	0.08	0.24	0.151	0.148	0.133

**Table 6:** ANOVA results and descriptive statistics of PFW indicators by age (Mean ± SD).

Age Group	WasteWg (g)	WWP (g)	WWF (g)	WWC (g)	WWE (kcal)
2 years	10.85 ± 22.10	0.45 ± 1.30	0.33 ± 0.85	1.30 ± 3.05	10.42 ± 24.20
3 years	11.02 ± 24.70	0.46 ± 1.33	0.34 ± 0.87	1.32 ± 3.08	10.55 ± 24.60
4 years	11.12 ± 25.40	0.46 ± 1.34	0.34 ± 0.88	1.33 ± 3.10	10.61 ± 24.80
5 years	11.41 ± 25.90	0.47 ± 1.37	0.35 ± 0.89	1.35 ± 3.13	10.73 ± 25.10
6 years	10.74 ± 24.60	0.45 ± 1.32	0.33 ± 0.86	1.31 ± 3.06	10.50 ± 24.50
7 years	11.50 ± 26.20	0.48 ± 1.36	0.36 ± 0.89	1.36 ± 3.12	10.80 ± 25.00
F(df = 5,14286)	0.59	<0.01	<0.01	<0.01	<0.01
*p*-value	0.71	1.00	1.00	1.00	1.00

**Table 7:** Two-way ANOVA results and descriptive statistics of PFW by age and gender type (Mean ± SD, g).

MealWg	2 Years	3 Years	4 Years	5 Years	6 Years	7 Years
Boys (Mean ± SD)	110.43 ± 63.54	110.23 ± 63.52	110.21 ± 63.48	110.17 ± 63.42	110.20 ± 63.46	110.20 ± 63.45
Girls (Mean ± SD)	110.23 ± 63.51	110.23 ± 63.51	110.26 ± 63.53	110.24 ± 63.50	110.24 ± 63.49	110.23 ± 63.55
**WasteWg**	**2 Years**	**3 Years**	**4 Years**	**5 Years**	**6 Years**	**7 Years**
Boys (Mean ± SD)	10.5 ± 22.3	13.0 ± 25.6	13.4 ± 26.0	11.2 ± 25.1	9.0 ± 21.8	13.0 ± 26.9
Girls (Mean ± SD)	10.1 ± 21.9	9.5 ± 23.4	9.1 ± 22.7	11.6 ± 25.5	11.9 ± 25.0	10.8 ± 25.8
**Two-way ANOVA MealWg**	**F (df)**				***p*-value**	**Partial η^2^**
Age	F(5,14280) = 0.001				1.000	0.000
Sex	F(1,14280) = 0.000				1.000	0.000
Age × Sex	F(5,14280) = 0.001				1.000	0.000
**Two-way ANOVA WasteWg**	**F (df)**				*p*-value *	**Partial η^2^**
Age	F(5,14246) = 0.83				0.531	0.000
Sex	F(1,14246) = 1.65				0.199	0.000
Age × Sex	F(5,14246) = 8.08				<0.001	0.005
Covariates	F(1,14246) = 1692.79				<0.001	0.106

* Vectorial tests significant at the 0.001 level.

**Table 8:** Communalities.

Indicator *	MealWg	WasteWg	MWP	MWF	MWC	MWE	WWP
Initial	1.000	1.000	1.000	1.000	1.000	1.000	1.000
Extraction	0.665	0.862	0.794	0.656	0.754	0.997	0.836
**Indicator**	**WWF**	**WWC**	**WWE**	**FLDRP**	**FLDRC**	**FLDRL**	**FLDRE**
Initial	1.000	1.000	1.000	1.000	1.000	1.000	1.000
Extraction	0.865	0.889	0.989	0.819	0.868	0.881	0.989

* Extraction Method: Principal Component Analysis.

## Data Availability

The anonymized dataset (14,292 observations), food composition tables, and SPSS syntax files used for descriptive and PCA analyses are available on reasonable request from the corresponding author. The data are not publicly available due to privacy and ethical restrictions.
